# Supplementary material for: Splice-Junction-Based Mapping of Alternative Isoforms in the Human Proteome
Source: Cell Rep. Author manuscript; Available in PMC 2020 Jan 15. (PMC6961840; doi:10.1016/j.celrep.2019.11.026)
Supplement: 2 [file NIHMS1546469-supplement-2.zip › DF1/PXD010154/Heart/Heart_9_TTN_SNCTVSVHVSEPATIVEKPESIK.pdf]

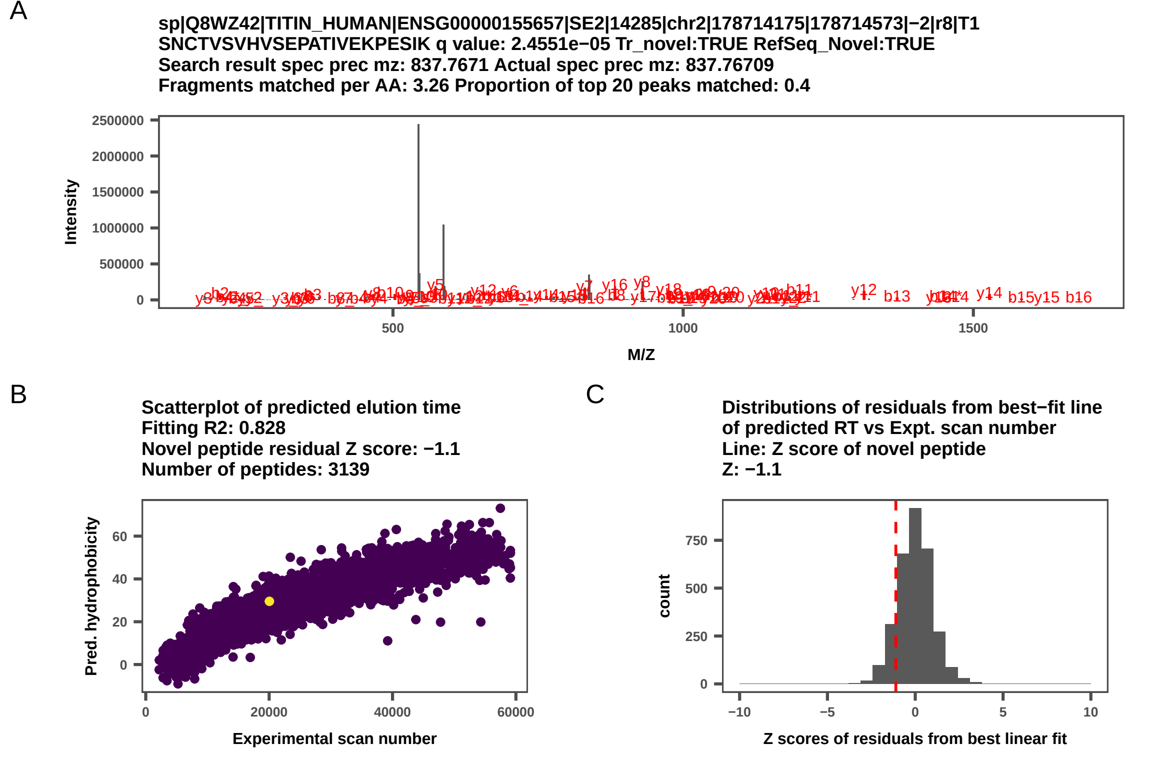
A
sp|Q8WZ42|TITIN_HUMAN|ENSG00000155657|SE2|14285|chr2|178714175|178714573|−2|r8|T1
SNCTVSVHVSEPATIVEKPESIK q value: 2.4551e−05 Tr_novel:TRUE RefSeq_Novel:TRUE
Search result spec prec mz: 837.7671 Actual spec prec mz: 837.76709
Fragments matched per AA: 3.26 Proportion of top 20 peaks matched: 0.4
Intensity
2500000
2000000
1500000
1000000
500000
0
M/Z
500
1000
1500
y5
y7
y16
y8
y12
b13
y14
b15
y15
b16
B
Scatterplot of predicted elution time
Fitting R2: 0.828
Novel peptide residual Z score: −1.1
Number of peptides: 3139
Pred. hydrophobicity
60
40
20
0
Experimental scan number
0
20000
40000
60000
C
Distributions of residuals from best−fit line
of predicted RT vs Expt. scan number
Line: Z score of novel peptide
Z: −1.1
count
750
500
250
0
Z scores of residuals from best linear fit
−10
−5
0
5
10
